# Genetic Testing between Private and Public Interests: Some Legal and Ethical Reflections

**DOI:** 10.3389/fpubh.2018.00008

**Published:** 2018-01-31

**Authors:** Judit Sándor

**Affiliations:** ^1^Center for Ethics and Law in Biomedicine, Central European University, Budapest, Hungary

**Keywords:** genetic testing, public vs private interface, hereditary diseases, intellectual property, health economics, human DNA

## Abstract

In Europe, there is a wide variety of genetic tests that various private companies offer to patients or to consumers. More and more people have become curious about their genetic predisposition and susceptibility. Most public health-care systems, however, are not adequately prepared for responding to these new demands and to the results of these genetic tests as, quite often, there is no available therapy for the identified genetic condition. This discrepancy between the newly emerging expectations and the insufficient responses contributes to a further rift between the public and private sectors of health care. Individual genetic test results may also trigger the need for personalized medicine and may open up a competition between the two fields in offering further genetic tests and medical exams. Pro-active patients may need a different kind of information on genetic tests and their implications. In this context, how should the public health system deal with the challenges of private testing? Will private genetic testing transform health care from a solidarity-based system to an individualistic one? In this paper, I would like to explore the emerging legal and ethical issues related to genetic testing and the relevant legal framework that has developed so far. In the conclusion, I will examine the possibilities of further legal development.

## Introduction

What is the role of the public and private sector in providing better care and information for patients based on genetic tests? Should direct-to-consumer (DTC) testing be controlled or should regulators give a wide scope of individual autonomy? What is the threshold of reliability? If further collection of the population based genetic data is necessary, how to balance between public and private interests? Could private companies represent public interests? Just looking at the long list of these questions, one can see that the core of the ethical and legal dilemmas today is to what extent can be considered genetic data as private and to what extent it can be used for public purposes? Furthermore, do personalized medicine and pro-active patients require some changes in the content of informed consent? It seems that people are more and more curious about their genetic data, and in this process, testimonies of the celebrities played an important role.

## The Jolie Effect in Personalized Medicine

Angelina Jolie made an important statement and even published an article in the May 14, 2013 issue of *The New York Times*, in which she started out with an account of the decade-long fight her mother had waged against ovarian cancer, at the end of which Jolie’s children had lost their 56-year-old grandmother. The article then took a startling turn, when the movie star—considered by many as one of the most beautiful women in the world, who at the peak of her career seemed to be in perfectly good health—informed the public that during the past few months her breast tissues had been surgically removed (in medical terminology, she had a double mastectomy). Although she had not actually been diagnosed with cancer, she decided to have a series of radical, prophylactic operations, which she intended to follow up with the surgical removal of her ovaries, pledging to publish the regimen of the medical procedures on a webpage.

To protect her family and children, she has opted for a preventive surgery to eliminate the chances of developing a cancer that has not even been diagnosed—and in doing so she has provoked a heated debate. Jolie’s coming-out, along with the public debate it has sparked, has ramifications in a range of issues as diverse as the boundaries between healthiness and illness, the precise definition of prevention, the financing of, and access to, genetic tests and the possible evaluation of the results, the expectations about the female body, the media representation of the celebrity world, the yawning gap between the health-care options available to the rich and the poor, as well as the prejudices and stigmas associated with cancer.

Although genetics promises to be the science of the future, this is the first time that a genetic test has driven a patient (or a potential patient, rather) to have a series of preventive medical procedures as radical as this. This is true even when we take into account that prophylactic mastectomies are much more frequent in the US: the American women’s decision is made easier by the fact that in such cases the cost of breast-reconstruction comes under health insurance coverage according to a federal law passed in 1998.

In recent years, considerable advances have been made in the field of genetics: now it has become possible to conduct detailed *BRCA* tests that can examine all the mutations. Located on chromosome no. 17, the gene *BRCA1* has approximately 1,180 different variants, with each one of them being capable of disrupting the proper functioning of the gene. Thanks to the huge genetic databases created by the private company Myriad Genetics of Utah, the risk factors can be identified with a relatively high certainty. In other words, it has become possible to estimate the probability of an individual’s developing ovarian cancer or breast cancer before the age of 70. According to the test results, Jolie belonged to the group with the highest risk factors. (Before the operation, the probability of eventually her developing breast cancer was as high as 86%, while she also had a 50% chance of falling ill with ovarian cancer.) The mutation discovered in her gene had already been found in her mother who died prematurely of ovarian cancer. For this reason, a number of people suggested psychological trauma or a pathological fear of cancer as possible motives for her decision. Actually, the truth is, she made a very rational decision: Jolie did not want to run the risk being diagnosed with a serious illness already at an advanced stage.

## The Health-Conscious Celebrity

The public seems to expect that celebrities conceal or deny their personal weaknesses and imperfections up to the hilt. It was this tacit agreement that Jolie has abrogated by going public with the news of her willfully chosen mastectomy—a medical procedure that so far has always been a hushed-up and divisive method of protection. But there is more. Jolie’s case questions the existence of a firm division between healthiness and sickness, considering that one would be hard-put to find the right expression to describe her status in this story: is she ill? Is she a patient? Is she a conscious consumer going out shopping on the health market? Is she a spokesperson for biotechnological advances? The expressions “patient” and “ill” would suggest that Jolie has been the forbearing sufferer who meekly submitted to a medical procedure prescribed by her doctor, instead of taking control of the situation, of planning and executing her own course of action.

But in fact that was not what happened here: Angelina Jolie overturned the traditional relationship between doctors and patients; one that is characterized by the latter’s vulnerability, helplessness, and dependence. In this case, it has been the patient who dictated the terms: she has availed herself of the medical services, had her tests, asked for consultation, weighed her options, and made her own decision. All this is in stark contrast with the helplessness of a cancer patient who, after much anxiety and long procrastination, finally makes up her mind to ask for an appointment with a specialist, patiently waits for weeks until her turn, goes to have a mammography, waits again to be scheduled for biopsy and then surgery; begs and bargains, and then, after a series of various operations and chemotherapy, she discovers that the idea of having reconstructive surgery will always remain an unrealizable dream for her. Before this, we saw mastectomy, or an invasive surgery, as a sacrifice ordered by the doctor and rendered by the patient, while considering the invasive medical action as the stigma of a serious illness, cancer, the antechamber of death, rather than the means to secure our life and health.

Jolie’s attitude has ushered in the prototype of a new category of patients—proactive consumers who plan preventive measures, who wish to extend and enjoy life, who consider genetics not as an act of fate but as an opportunity—in other words, conscientious biotechnological consumers. Cancer patients carry multiple disadvantages, as the stigma of a serious illness is usually accompanied by the stigma of poverty also. (Faced with prolonged unemployment, even middle-class patients can find themselves in severe financial straits. Genetics can overwrite our notions about the discriminative nature of illnesses.) However, at the moment, this remains closely bound up with the exclusivity and high price-tag of the tests.

Breast cancer is the second leading cause of death among American women; it is estimated that in the US the genetic testing designed to reveal the presence of BRCA1 gene is performed on about 250,000 women annually. The basic version of the BRCA test costs between 3,000 and 4,000 dollars—and the simpler tests do not even cover all the potentially fatal mutations. Studies of the mutant genes responsible for the development of breast cancer get more and more accurate and reliable as the number of the tested mutants increases. To evaluate the tests, one needs huge databases, which can only be set up by private biotechnological companies and clinics where extensive data collection has been carried out for decades. The precise interpretation of the genetic tests results also requires a great amount of work and money. The crucial questions that now must be asked are as follows: how much should be paid for these still highly expensive tests and who should be paying for them? How should the price of the tests be determined, and by whom, so as to ensure that the actors of this market stay in business on the one hand, and do not exploit their monopoly on information on the other? And in a broader context: to what extent is it permissible that the patients’ prospects to live a long and healthy life be commensurate with their financial circumstances?

BRCA1 and BRCA2 are human genes that produce tumor suppressor proteins. These proteins have a vital role in repairing the damaged DNA. When one these genes does not function correctly, DNA damage may not be repaired properly, and as a consequence, cells may develop additional genetic mutations that can lead to cancer.

According to the US National Cancer Institute, specific inherited mutations in BRCA1 and BRCA2 increase the risk of female breast and ovarian cancers, “and they have been associated with increased risks of several additional types of cancer. Together, BRCA1 and BRCA2 mutations account for about 20–25% of hereditary breast cancers and about 5–10% of all breast cancers. In addition, mutations in BRCA1 and BRCA2 account for around 15% of ovarian cancers overall.”^1^

Furthermore, “a BRCA1 or BRCA2 mutation can be inherited from a person’s mother or father. Each child of a parent who carries a mutation in one of these genes has a 50% chance of inheriting the mutation. The effects of mutations in BRCA1 and BRCA2 are seen even when a person’s second copy of the gene is normal.”^2^

The US National Cancer Institute provided comparative statistical figures on the significant differences between the occurrence of cancer in the general population and cancer in the population that has some genetic predisposition. “About 12% of women in the general population will develop breast cancer sometime during their lives. By contrast, according to the most recent estimates, 55–65% of women who inherit a harmful BRCA1 mutation and around 45% of women who inherit a harmful BRCA2 mutation will develop breast cancer by age 70 years.”^3^

The legal approach to all these problems has previously been anchored to the instrument of patent protection: the standard practice of private companies specializing in genetic technology so far has been to obtain patent protection for the DNA sequence they have successfully isolated—this has been the corner stone of their business model. It is a small wonder, then, that nowadays the debates about studies and patents associated with predisposing genes for breast cancer and ovary cancer heat up. In early 2010, a total of 19 plaintiffs, including physicians, researchers, patients, the American College of Medical Genetics, the American Society of Clinical Pathologists, and several researchers at the University of Pennsylvania, formed an unprecedented joinder of parties to file a case against the US Patent Office and Myriad Genetics. In its claim against the company holding the patent, the American Civil Liberties Union (ACLU) argued that genes are the products of nature, rather than of human beings, and thus fall outside the jurisdiction of patent law. In addition, the strict legal protection, ACLU claimed, severely limits the researchers’ work and is, therefore, in violation of the patients’ rights. Representatives of Myriad, the company holding the patent rights, asked the Court to reject the petition, claiming that their company was the first one to isolate these genes within the DNA and, therefore, has acquired the right to place them under patent protection. According to the plaintiffs, through its practice, Myriad keeps the prices high and prevents the patients from checking the test results.

## The Proactive Patient Model

According to the Transparency Market Research,^4^ the annual growth rate of the gene sequencing were 17.5% in 2016. While genetics promises to be the science of the twenty-first century, its results often lead to therapy or prevention, which lie in the obsolete surgical techniques of the twentieth century, as in fact demonstrated by Jolie’s case, too (1).

It can hardly be argued though, that genetics, and our knowledge of our own genes, in itself has an emancipatory effect. It is quite obvious that in these difficult questions only the well-informed and active patient can make a carefully weighed decision. As it has been demonstrated by a number of examples already, the model of a patient condemned to a role of passive acceptance is no longer a given in the field of genetics. (People suffering from the so-called Canavan disease have, for example, organized and supported the genetic study of their own illness, but there have been numerous other self-organized particular bio-society groups all over the world, who chose to take control of their own fate.) The case of Angelina Jolie certainly fits into this trend. The effect that the coming out of such a world-renowned celebrity has exerted on the knowledge of medicine, genetic tests, and the expectations of health care of millions of women all over the world could easily have been more profound than a myriad of official health campaigns put together with scientific thoroughness. The reverberations of her announcement are still unclear at this point; but we may certainly hope that it will inspire more and more healthy women and men to act as conscious biotechnological consumers; it may convince them to take their genetic lottery tickets and cash them. This conscious behavior may shake up and reorganize the balance of power in the field of health care, resulting in a number of positive developments. The less fortunate outcome of Jolie’s act may be the increased commercialization of biotechnology, which, through the extraordinary publicity, could lead to the higher pricing of genetic tests, further widening the gap between patients of different financial status.

Genetic data can be used for a number of purposes, including the establishment of paternity or personal identity, genealogical research, and the confirmation of exceptional talent. And as the full potentials of genetic testing became ever more apparent, people’s imagination began to go wild. In Italy, for example, it prompted huge media interest and full television coverage when members of a family by the name of Merisio were subjected to oral mucosal swabs to prove their claimed genetic descent from the painter Caravaggio. But genomics’ glamorous association with fame had already begun in 2010. This was when the public found out that the first woman with a known identity to have her full genome analyzed was the famous American actress, Glenn Close. Compared to the $500 million that such a comprehensive procedure had cost at the beginning of the Human Genome Project, the price has now climbed down to $48,000, thanks to new advances in technology.

For a large number of people, genetic data represent the most guarded private secrets, in view of the fact that they offer a glimpse of not only possible current illnesses but also of risk factors and personal susceptibility. By contrast, many people do not feel the need to conceal any of their genetic data. The Personal Genome Project (PGP), which involved the participation of a large number of volunteers, was conducted in a completely open manner. PGP has encouraged us to rid ourselves of suspicions and prejudices fixed in our minds and to contribute to a monumental project of genetic research by giving our name and donating our medical data. The findings were later made available to the participants themselves. The home page of the project features 10 researchers, all in apparent good health, who reveal their genetic data to the public. They have no reason to conceal that they suffer from myopia or asthma, or that they sometimes have trouble sleeping because of too much traveling.

As a result of what is known as trans-generational effect of genetic information, the findings of genetic testing may also be of concern to people other than the one tested. As Jolie’s suspicions were originally raised by her mother’s illness, it is probable that Jolie’s children will also have to go through genetic testing at some point. Their chances of making the right choices could even be better than their mom’s, considering that the conclusions in their case will have been drawn from data provided by two consecutive generations. We can only hope that by that time they will have better options than invasive surgery.

## Does Genetic Testing Require a New Privacy Approach?

Article 10 (2) of the Oviedo Convention states clearly that “[e]veryone is entitled to know any information collected about his or her health. However, the wishes of individuals not to be so informed shall be observed.”^5^

In connection with genetic research, there has emerged a growing demand for the establishment of genetic databanks operated in national or other institutional frameworks. These genetic databanks call for special regulations even if they are involved only in data storage and data management, or operate as tissue banks. Genetic tests provide specific challenges to the protection of private life (2).

Twenty years ago, it was a widely shared opinion that anonym data could guarantee the highest level of data protection for genetic information. Later on, however, experts identified a wide array of ethical and legal problems. One of the issues is that fully anonymized genetic data cannot be compared with the health data of the specific patient and, consequently, the data are not very useful for scientific research. Anonymization is also problematic for the patients/research participants, since it would be impossible for them to receive feedback on their data provided, due to the anonymization itself.

One difficulty that laws on biotechnological advances always face is how to balance between legal and scientific definitions of certain terms. While science may develop comfortably without laying down strict legal definitions of the terms “biobank” or “genetic data,” lawmaking requires these definitions to be exact and understood unequivocally, even across the related legal instruments. This, however, brings about the problem of inflexibility: as biotechnological inventions develop, such as in the fields of cloning or stem cell research, new discoveries and technologies might not fit the old definitions.

Genetic information has brought about specific legal problems in data processing, as well as in data protection (3). The scope of genetic data itself is difficult to define. Certain family-related medical data should be viewed to belong to this category alongside susceptibility factors or *monogenic* disorders (caused by the defect or deficiency of a single gene). The accuracy of and potential for using these data are highly variable. Information on any hereditary disease has a serious impact on the lives and decisions of family members who may not have wanted to subject themselves to test examinations.

Genetic data have their implications on lifestyles, routines, selection of spouses, decisions to have children, career preferences, and even the learning ambitions of people. Whoever decides to become acquainted with these parameters has the opportunity to become informed within the limits that the health-care system can offer. In legal terms, the question is rather how the right of disposal over genetic data can be preserved (4).

The main argument behind genetic exceptionalism is that personal genetic data should be processed under special legal guarantees, as genetic data can be considered not only as a simple health-care data^6^ but it can be also used for identification purposes. In comparison with conventional medical data, another major difference is that genetic data do not only refer to the medical status of the examined person, but may reveal the medical characteristics of the affected family members or even children about to be born. Therefore, it can be claimed that medical data are generated without the persons concerned knowing about the existence of the data.

A key condition of the legislative act on data protection is that any data processing should be bound to a specific purpose. Thus, personal data may be processed only for stated purposes, toward the proper exercise of rights and fulfillment of obligations. Data processing should comply with the given purpose in all its phases. Furthermore, only such personal data can be managed that are essential to accomplish the given objective of data management, suitable for the purpose stated, but only to the extent and for the duration actually required for the accomplishment of the underlying objective. So in strict interpretation medical data—not to mention genetic information—generated for medical purposes and—for instance—forming parts of a given documentation, cannot be disclosed in their original forms to insurance companies, but may be revealed only to the extent they are absolutely necessary for the insurance activities.

Many distinguished social scientists, even bioethicists^7^ [see Ref. (5)], however, did not subscribe for these special claims associated with genetic regulation. In order to challenge the legitimacy of the particular regulation, they pointed out that many characteristics of the data, predictive, particular sensitive can be also seen at other types of health-care data.

Legal scholars are also hesitant to jump to enact special legal provisions to genetic data (6). Usually health-care professionals, especially those who work in the field of biobanks favor *tabula rasa* and eager to seek specific law that governs their situation so that they do not have to worry about often vague legal interpretations of general legal norms on data protection.

It follows from the spirit of data protection norms that even rules on biomedical research have to be developed in order to respond to different kinds of uses of genetic data. For instance, consent for collection and for specific use of genetic data is a precondition for the research. But then legal policy should differentiate whether the research material still carries personal information or not. When human tissue samples are anonymized in the sense that it is no longer possible to trace back the identity of the person from whom these tissues have been harvested then legal concerns are minimized to governing the tissue management.

In the case of *S. and Marper*,^8^ the European Court for Human Right first time made a decision on the retention of the genetic data in the criminal justice system. The Court found that there had been a violation of Article 8 of the European Convention for the Protection of Human Rights and Fundamental Freedoms, stating:

“[…] the Court finds that the blanket and indiscriminate nature of the powers of retention of the fingerprints, cellular samples and DNA profiles of persons suspected but not convicted of offenses, as applied in the case of the present applicants, fails to strike a fair balance between the competing public and private interests and that the respondent State has overstepped any acceptable margin of appreciation in this regard. Accordingly, the retention at issue constitutes a disproportionate interference with the applicants’ right to respect for private life and cannot be regarded as necessary in a democratic society.”^9^

Although this case was dealing with the genetic data in the forensic context, nevertheless, its legal reasoning is an important step in the legal thinking on genetic data as this Court decision realized that not only genetic data but also genetic samples should be protected under privacy and data protection norms.

## The Necessity of New Guidelines for Informed Consent

Personalized medicine and DTC genetic testing would demand a more active role from the patient in health care. When patients are informed about genetic tests and treatment from the web, subsequent information, provided by the health-care professionals should be different than before. On the one hand, doctors may save some time by leaving the explanation of basic health information to the patient’s internet search, in the same time, information found on the web may have been better explained. Doctor and patient may become really good partners in health care, but when information found on the web is misleading or the doctor disagrees with the patients’ choices then an additional attempt has to be made by providing arguments for the test or treatment that he finds medically appropriate. With genetic consultation and by vigorous monitoring of genetic tests, there would be lots of advantages to this new model. Patients would be more conscious of their chosen life style and would be better educated in general about various genetic and non-genetic components of their illness. Nudges might be still necessary in many fields. Doctors should express their doubts when patients insist to a test that is medically not necessary or not appropriate. Medical indifference can be harmful just as much as total ignorance of the patients’ choices or preoccupation with certain familiar or genetic risks. Personalized health care may require new ethical and legal norms to ensure a better communication between the doctor and the patient and a better quality of health care. Furthermore, public and private genetic services should be harmonized in terms of quality and scope.

In recent decades, legal rules of the informed consent have tended to follow the individual information model to a growing extent: it means that information is provided only to the patient provided that s/he is capable to act, while family members may be informed only if the patient expressly consents so. It seems, however, that genetic information will potentially re-establish the role of information provision to the family (7). In the case of certain diseases, there are just a few people to undergo routine medical screening. Yet, when such a disease appears in a family, it is regarded as a reason for family members to take screening examinations and prevention more seriously, especially when the disease is known to be hereditary (8). With the enlarging scope of genetic knowledge, more and more diseases are likely to involve the obligation of physicians to warn the family members (9).

In the *Munro v. Regents of University of California* case,^10^ for instance, the Court of Appeal of the State of California claimed the defendant physician to be responsible for failing to conduct the Tay-Sachs test. In the *Safer v. Pack* case,^11^ the court ascertained the liability of the physician for the failure to provide information when he did not warn the relatives of a patient who had been treated for a known hereditary disease of the associated risks. In the *Pate v. Threlkel*^12^ case, the Court of Appeal of the State of Florida also confirmed the obligation of the physician to warn the children of the patient.

In case of genetics, the individual consent model is also often challenged (10). It follows from the recognition of the interest of several third parties in genetic data that justification of the individual consent model is problematic or at least requires some adjustments.

## International Legal Norms

In the field of biomedicine, several special organizations within the *United Nations* issued declarations. UNESCO has a priority in standards settings in the field of bioethics within the UN. UNESCO adopted three major declarations, two of them had specific content in the field of genetics: the Universal Declaration on the Human Genome and Human Rights^13^ and the International Declaration on the Human Genetic Data.^14^ Article 4 of the International Declaration on Genetic Data grants a special status to the human genetic data. This approach has been often criticized calming that this document represents genetic exceptionalism. I think, nevertheless, it was important to initiate thinking on genetic data and to provide reasons why genetic data should be protected. According to the Declaration, genetic data should be protected because they can be “predictive of genetic” dispositions concerning individuals; furthermore they “may have a significant impact on the family, including offspring, extending over generations, and in some instances on the whole group to which the person concerned belongs.” Genetic data may contain information the significance of which is not necessarily known at the time of the collection of the biological samples; and they may have cultural significance for persons or groups.^15^

The Council of Europe played a very important role in adopting key international norms in the field of biomedicine. The most important one is the *Convention for the Protection of Human Rights and Dignity of the Human Being with regard to the Application of Biology and Medicine: Convention on Human Rights and Biomedicine*^16^ (signed in Oviedo in 1997), and its four additional protocols: the *Additional Protocol on the Prohibition of Cloning Human Beings*^17^ of 1998, the *Additional Protocol concerning Transplantation of Organs and Tissues of Human Origin*^18^ of 2002, the *Additional Protocol concerning Biomedical Research*^19^ of 2005, and *Additional Protocol concerning Genetic Testing for Health Purposes*^20^ of 2008, which is the most relevant international document in the field of genetics. The success of these norms can be seen by the increasing reference to them by the European Court of Human Rights.

While general data protection is provided in the field of protecting genetic data, the most specific legal instrument in Europe was adopted in 2006: the Recommendation Rec(2006) 4 of the Committee of Ministers to Member States on Research on Biological Materials of Human Origins. The extension of the language of human rights to the field of human tissues is clear in the stated objective of the Recommendation. As such, Article 11 of the Recommendation harmonizes the rules on the research on human beings with the rules on human tissues. “An intervention should only be carried out to obtain biological materials for storage for research purposes if it complies with the Additional Protocol concerning biomedical research ….”^21^

The Additional Protocol to the Convention on Human Rights and Biomedicine concerning Genetic Testing for Health Purposes^22^ applies to tests, which are carried out for health purposes, involving analysis of biological samples of human origin and aiming specifically to identify the genetic characteristics of a person, which are inherited or acquired during early prenatal development. The Protocol does not apply to genetic tests carried out on the human embryo or fetus; to genetic tests carried out for research purposes.

Similarly to the Oviedo Convention, its Protocol also emphasizes the primacy of the human being. “The interests and welfare of the human being concerned by genetic tests covered by this Protocol shall prevail over the sole interest of society or science.”^23^ In Article 4, the principles of non-discrimination and non-stigmatization are laid down. “(1) Any form of discrimination against a person, either as an individual or as a member of a group on grounds of his or her genetic heritage is prohibited. (2) Appropriate measures shall be taken in order to prevent stigmatization of persons or groups in relation to genetic characteristics.”^24^

One of the most interesting provisions of the Protocol can be found in Article 7, which prescribes “individualized supervision” by stating that “a genetic test for health purposes may only be performed under individualized medical supervision.”^25^ Although exception may be allowed are allowed but “such an exception may not be made with regard to genetic tests with important implications for the health of the persons concerned or members of their family or with important implications concerning procreation choices.”^26^

As to the European Union law, the Charter of Fundamental Rights of the European Union of 2000 should be mentioned. The importance of the Charter cannot be underestimated, it is the Bill of Rights of the European Union, and it lists fundamental rights shared by European Union member States. In the field of bioethics, especially Article 1 on human dignity, and more specifically, Article 3 on the right to the integrity of the person are of great relevance. As to the sources of secondary legislation, the following documents are relevant: regulation (EC) No 45/2001 of the European Parliament and of the Council of 18 December 2000 on the protection of individuals with regard to the processing of personal data by the Community institutions and bodies and on the free movement of such data, the Directive 98/44/EC of the European Parliament and of the Council of 6 July 1998 on the legal protection of biotechnological inventions, and finally, Directive 95/46/EC of the European Parliament and the European Commission on the protection of individuals with regard to the processing of personal data and on the free movement of such data.^27^ From 2018, data protection norms will be further strengthen when the regulation on data protection^28^ will enter into force.

Finally, one should also mention the opinions and statements of the European Group on Ethics in Science and New Technologies (EGE). It made highly authoritative texts in questions related to the ethical aspects of umbilical cord blood banking (Opinion No. 19.)^29^; and on the ethical aspects of human tissue banking (Opinion no 11.). Furthermore in 2016, EGE also made a statement on gene editing.^30^

## Conclusion

In the field of the application of genetic knowledge, legal issues are mostly focused on the problem of how new genetic information affects our basic human relations, family ties, decisions over the reproduction, insurance, employment, and intellectual property. To be more precise, how should genetic information be qualified, what kinds of rights can be established on this knowledge, who should hold this knowledge, and who is to control this intrinsically individual, wide-ranging information that can easily be obtained by others? The fine-tuning and detailed scrutiny of our biological and genetic view on humans, as well as the mapping of the entire human genome, originate from a generally understandable scientific drive, and at first sight it produces mostly neutral information. Serious ethical and legal dilemmas arise especially when this knowledge is applied in a broader social context.

Now, almost 15 years after, the landmark research on the Human Genome—perhaps in a less passionate manner than at the beginning of the twenty-first century—we can better see the use of various tests but we are facing a new dilemma, how to preserve the European solidarity based health care, but also how can we respect individual choices in genetic testing. As we have seen the legal framework is still sporadic, and although basic human rights and data protection aspects of the genetic testing are regulated the connection between public and private sectors and using genetic tests from one sector to another needs more reflection. In the future, hopefully not only celebrities can benefit from the advances of genetic testing and with a more nuanced interpretation of genetic test genetic information will no longer be interpreted as destiny.

The complicated relation between private genetic services and public health genomics has many aspects. The more genetic test results will be available, the more we know about the treatment of the genetically linked diseases. As it follows, there are collective dimensions of the private tests as well, provided that there are scientific publications about the results and methods of testing and databanks follow the same rules. As it follows regulation in this field would guarantee not only patients; rights but also the validity and comparison of the data. In the field of genetics, there are many serious but rare conditions that would require an international co-operation both in terms of testing, as well as finding personalized care.

## Author Contributions

All aspects of manuscript completed by corresponding author JS.

## Conflict of Interest Statement

The author declares that the research was conducted in the absence of any commercial or financial relationships that could be construed as a potential conflict of interest. The reviewer MD and the handling editor declared their shared affiliation.

## Footnotes

^1^National Cancer Institute, BRCA1 and BRCA2: Cancer Risk and Genetic Testing, available at https://www.cancer.gov/about-cancer/causes-prevention/genetics/brca-fact-sheet, last accessed on August 25, 2017.

^2^Help Me Understand Genetics Inheriting Genetic Conditions, Reprinted from https://ghr.nlm.nih.gov/ Lister Hill National Center for Biomedical Communications, U.S. National Library of Medicine, National Institutes of Health, Department of Health and Human Services, Published July 11, 2017

^3^National Cancer Institute, *op. cit*.

^4^Transparency Market Research, Global Genomics Personalized Health Market: Snapshot, available at http://www.transparencymarketresearch.com/personalized-genomics-market.html, last accessed on August 25, 2017.

^5^Council of Europe Treaty Series (CETS) No. 164; the official text of the document is available at https://rm.coe.int/168007cf98.

^6^In the sense as it is treated under Article 8 of the of the European Parliament and Council Directive 95/46/EC of 24 October 1995 on the Protection of individuals with regard to the processing of personal data and on the free movement of such data.

^7^For example, Søren Holm challenged genetic exceptionalism already in 1997.

^8^*S. and Marper v. United Kingdom*, ECtHR, Applications no. 30562/04 and 30566/04, judgment of December 4, 2008, official text available at https://rm.coe.int/168067d216.

^9^*Ibid*., 125.

^10^No. B037779. Court of Appeals of California, Second Appellate District, Division Seven. November 16, 1989.

^11^Superior Court of New Jersey, Appellate Division,1996 291 N.J. Super A.2d 1188

^12^*Pate v. Threlkel* 661 So 2d 278 Fla. (1995).

^13^The Universal Declaration on the Human Genome and Human Rights was adopted unanimously and by acclamation at UNESCO’s 29th General Conference on 11 November 1997. The following year, the United Nations General Assembly endorsed the Declaration.

^14^To address these concerns, the International Declaration on Human Genetic Data was adopted unanimously and by acclamation at UNESCO’s 32nd General Conference on 16 October 2003.

^15^Article 4 of the International Declaration on Genetic Data.

^16^Council of Europe Treaty Series (CETS) No. 164; the official text of the document is available at https://rm.coe.int/168007cf98.

^17^CETS No. 168; the official text of the document is available at https://rm.coe.int/168007f2ca.

^18^CETS No. 186; the official text of the document is available at https://rm.coe.int/1680081562.

^19^CETS No. 195; the official text of the document is available at https://rm.coe.int/168008371a.

^20^CETS No. 203; the official text of the document is available at https://rm.coe.int/1680084824.

^21^https://www.coe.int/t/dg3/healthbioethic/Activities/10_Biobanks/Rec%282006%294%20EM%20E.pdf.

^22^CETS No. 203, *op. cit*.

^23^*Ibid*., Article 3.

^24^*Ibid*., Article 4.

^25^*Ibid*., Article 7.

^26^*Ibid*.

^27^Final Report of the GeneBanC Project, *European Ethical-Legal Papers* No.17. (2009) Leuven, 15.

^28^Regulation (EU) 2016/679 of the European Parliament and of the Council of7 April 2016 on the protection of natural persons with regard to the processing of personal data and on the free movement of such data, and repealing Directive 95/46/EC (General Data Protection Regulation)

^29^http://europa.eu/rapid/press-release_IP-04-364_en.htm?locale=en last accessed: 10 July, 2017.

^30^https://ec.europa.eu/research/ege/pdf/gene_editing_ege_statement.pdf last accessed: 10 July, 2017.
